# Determining the optimal contrast-enhanced voiding urosonography technique for vesicoureteral reflux in children and adolescents: a systematic review and network meta-analysis

**DOI:** 10.3389/fped.2025.1472382

**Published:** 2025-04-25

**Authors:** Sha Hu, Yu Tian, Min He

**Affiliations:** Department of Ultrasound, West China Second University Hospital, Sichuan University/Key Laboratory of Obstetrics & Gynecology, Pediatric Diseases, and Birth Defects of the Ministry of Education, Chengdu, China

**Keywords:** vesicoureteral reflux, voiding cystourethrography, contrast-enhanced voiding urosonography, diagnostic performance, network meta-analysis

## Abstract

**Aim:**

To evaluate diagnostic combinations of imaging modalities and contrast agents for vesicoureteral reflux (VUR) in children.

**Methods:**

Studies were retrieved in PubMed, EMBASE, Cochrane Library, Web of science, and China National Knowledge Infrastructure (CNKI) until March 16, 2023. We used bivariate random-effects model and a frequentist model for meta-analysis. Surface under the cumulative ranking curve (SUCRA) was used to rank ceVUS protocols.

**Results:**

19 studies identified 4 diagnostic combinations. Contrast-harmonic ultrasound with SonoVue® (CHSV) had significantly lower sensitivity, specificity, and diagnostic accuracy than VCUG, although its negative predictive value was higher than VCUG. Contrast-harmonic ultrasound with Optison™ (CHOS) was comparable to VCUG across all diagnostic measures. SUCRA analysis favored CHOS, with probabilities of being the best for SEN, SPE, PPV, NPV, DOR, and DA at 50.9%, 16.6%, 42.6%, 57.4%, 36.2%, and 20.4%, respectively.

**Conclusion:**

Harmonic VUS with Optison™ may be the optimal protocol for VUR detection in children.

**Systematic Review Registration:**

https://www.crd.york.ac.uk/PROSPERO/view/CRD42023424510, identifier (CRD42023424510).

## Introduction

Vesicoureteral reflux (VUR), the retrograde flow of urine from the bladder into the ureter and pelvicalyectasis system (1), is the most common urinary tract abnormality in children and adolescents (2). The incidence of VUR in the general pediatric population is estimated to be approximately 17.2%. However, among children presenting with a urinary tract infection (UTI), the prevalence can increase significantly, with estimates ranging from 30%–40% (3). The potential impact of VUR on patient health is profound, especially in pediatrics. It is a major contributor to recurrent urinary tract infections and can lead to renal damage, posing a significant threat to long-term health and development in children and adolescents (4, 5). Therefore, accurate diagnosis is paramount in formulating effective treatment strategies and preventing potential complications (6).

Currently, various imaging techniques, such as voiding cystourethrography (VCUG) and direct radionuclide cystography (DRNC), can be used to diagnose VUR, among which VCUG is the gold standard for diagnosing VUR (7). The American Academy of Pediatrics revised its guidelines in 2011, advising against routine VCUG following the first febrile urinary tract infection in children for the diagnosis of VUR due to potential radiation exposure (8). Therefore, efforts have been made to find alternative diagnostic strategies for the detection of VUR in children and adolescents, as studies have shown that attention should be paid to the radiation exposure during VCUG (9).

Contrast-enhanced voiding urosonography (ceVUS) allows ultrasound to provide functional information using contrast agents (10). With technological advances, the availability of high-resolution equipment, and the production of second-generation US contrast agents, ceVUS is now advocated as a safe and radiation-free alternative diagnostic protocol compared with traditional methods for the detection of VUR, minimizing the exposure to ionizing radiation in children and adolescents (11–13). To date, two meta-analyses (7, 14) have evaluated the diagnostic performance of ceVUS in the detection of VUR in children and adolescents. According to the meta-analysis by Chua et al. (14), ceVUS using second-generation contrast agents with harmonic imaging has acceptable diagnostic accuracy. However, Yousefifard et al. (7) found that the diagnostic performance of ceVUS with first- and second-generation contrast agents for VUR was comparable and within an excellent range. More importantly, this meta-analysis (7) revealed that there are different ceVUS imaging modalities (e.g., conventional, harmonic and doppler-based VUS) and two types of second-generation contrast agents (i.e., SonoVue®/Lumason® and Optison™) in clinical practice, thereby leading to various diagnostic combinations of different ceVUS imaging modalities and two types of second-generation contrast agents. SonoVue® and Lumason® are essentially the same ultrasound contrast agents but are marketed under different names in various regions used to enhance ultrasound imaging quality.Although some studies have investigated the synergistic diagnostic performance of different types of contrast agents combined with different ceVUS imaging modalities (14). However, due to the lack of direct comparisons between different diagnostic combinations of different ceVUS imaging modalities and second-generation contrast agents, there is still no evidence of whether different diagnostic combinations differ in their overall diagnostic performance for the detection of VUR. Therefore, we conducted this network meta-analysis to comprehensively evaluate the differences between various diagnostic combinations of various ceVUS imaging modalities with different seconde-generation contrast agents for the diagnosis of VUR in children and adolescents.

## Methods

We performed this network meta-analysis according to the Cochrane Handbook for systematic reviewers (15). In addition, we reported this network meta-analysis according to the Preferred Reporting Items for Systematic Reviews and Meta-Analyses (PRISMA) extension for Diagnostic Test Accuracy (DTA) (16) and the Preferred Reporting Items for Systematic Reviews and Meta-Analyses (PRISMA) extension statement for reporting network meta-analysis (17). We have registered our study on PROSPERO with the registration number CRD42023424510.

### Literature search

We searched PubMed, EMBASE, Cochrane Library, Web of Science, and China National Knowledge Infrastructure (CNKI) for potentially eligible studies from the inception of each database to March 16, 2023. We developed search strategies using Boolean operators (AND, OR, NOT) combining the following terms and their analogs, including vesicoureteral reflux and voiding urography. Supplementary Table S1 summarizes the detailed search strategies for the targeted databases. We restricted our searches to human studies but did not restrict language or study design. In addition, we also checked additional studies by manually screening the references of previous meta-analyses.

### Selection criteria

Based on previous meta-analyses (7, 14), we designed the inclusion criteria for this network meta-analysis: including (1) patients under the age of 18; (2) patients diagnosed with VUR, irrespective of the presence or absence of other confounding factors including a neurogenic bladder, renal anomalies and so on; (3) the reference standard was VCUG; (4) studies reported the diagnostic performance compared ceVUS to VCUG in detecting VUR; and (5) data reported by studies could be converted into true positive (TP), false positive (FP), false negative (FN), and true negative (TN). We excluded studies based on the following criteria: (1) ineligible study design, such as case series, case reports, and conference abstracts; (2) VCUG was not used to diagnose VUR in the article; (3) the use of ceVUS protocol was not described; and (4) no data available for estimating required diagnostic measures.

### Study selection

After removing duplicate records, two independent authors selected eligible studies through the following steps. First, the two authors screened the titles and abstracts of all studies for initial eligibility assessment. Second, the same two authors independently screened the full texts of the studies retained from the first step to determine their eligibility. These two authors determined the final inclusion of each study through discussion.

### Data extraction

Two independent authors (Sha Hu and Yu Tian) extracted relevant data from each study using a pre-designed data extraction form, including first author name, year of publication, country, study design, sex ratio, average age, the number of the kidney-ureteral unit, indication for VUR investigation and details for ceVUS. Additionally, the same two authors extract or calculate the values for TP, FP, FN, TN of each ceVUS protocol to reconstruct the two-by-two tables. Discussion between the two authors (Sha Hu and Yu Tian) was used to resolve any disagreements.

### Quality assessment

Two independent authors (Sha Hu and Min He) assessed the overall quality of each study using the Quality Assessment of Diagnostic Accuracy Studies (QUADAS-2) (18). In the QUADAS-2 tool, the risk of bias must be assessed in four domains: patient selection, index testing, reference standard, process, and timing. Applicability needs to be assessed in three domains, including patient selection, index testing, and reference standards.

### Statistical analysis

We first performed pairwise diagnostic meta-analyses to evaluate the diagnostic performance of each ceVUS protocol for detecting VUR compared with VCUG. We chose a bivariate model to estimate pooled sensitivity (SEN), specificity (SPE), positive predictive value (PPV), negative predictive value (NPV), diagnostic accuracy (DA), and diagnostic odds ratio (DOR). The overall diagnostic yield of each protocol was assessed using the hierarchical summary receiver operating curve (HSROC) and the area under the cumulative curve (AUC). We used Cochrane's *Q* test to determine whether statistical heterogeneity was present and used *I^2^* statistics to quantify the level of statistical heterogeneity in this meta-analysis. Nevertheless, we chose the random-effects model for the meta-analysis because variations across studies cannot be eliminated in real-world settings. STATA 14.0 (STATA Corporation, Lakeway, Texas, USA) was used for all these statistical analyses.

To compare different ceVUS protocols, we used frequentist network meta-analysis with the “network” command in STATA 14.0 software according to the frequentist method. We performed the transitivity assessment by assessing the distribution of four major factors, including the year of publication, sample size, gender ratio, and mean age, across studies. Based on the transitivity assessment results, we knew it was appropriate to conduct a network meta-analysis without additional procedures for processing data. We used mean difference (MD) with corresponding 95% confidence interval (CI) to estimate the difference in SEN, SPE, PPV, NPV, DOR, and DA between different combinations of different ceVUS imaging modalities with different second-generation contrast agents. We did not assess global and local inconsistencies because this network meta-analysis has no closed loop. Furthermore, loop-closed inconsistency did not apply to this network meta-analysis. Therefore, we performed a network meta-analysis using the consistency model. In addition, we calculated the surface under the cumulative ranking (SUCRA) values to rank all available ceVUS protocols. SUCRA are calculated by summing the cumulative ranking probabilities for each possible rank and then normalizing this sum to provide a value between 0 and 1, where higher values indicate a greater likelihood of an intervention being among the best in the ranking. SUCRA values are a ranking mechanism and not the actual sensitivity and specificity percentages, where higher values indicate a better ranking among compared interventions. Finally, outcomes involving more than five eligible studies were examined for publication bias using Deeks' funnel plot.

## Results

### Literature search

**Figure 1:**
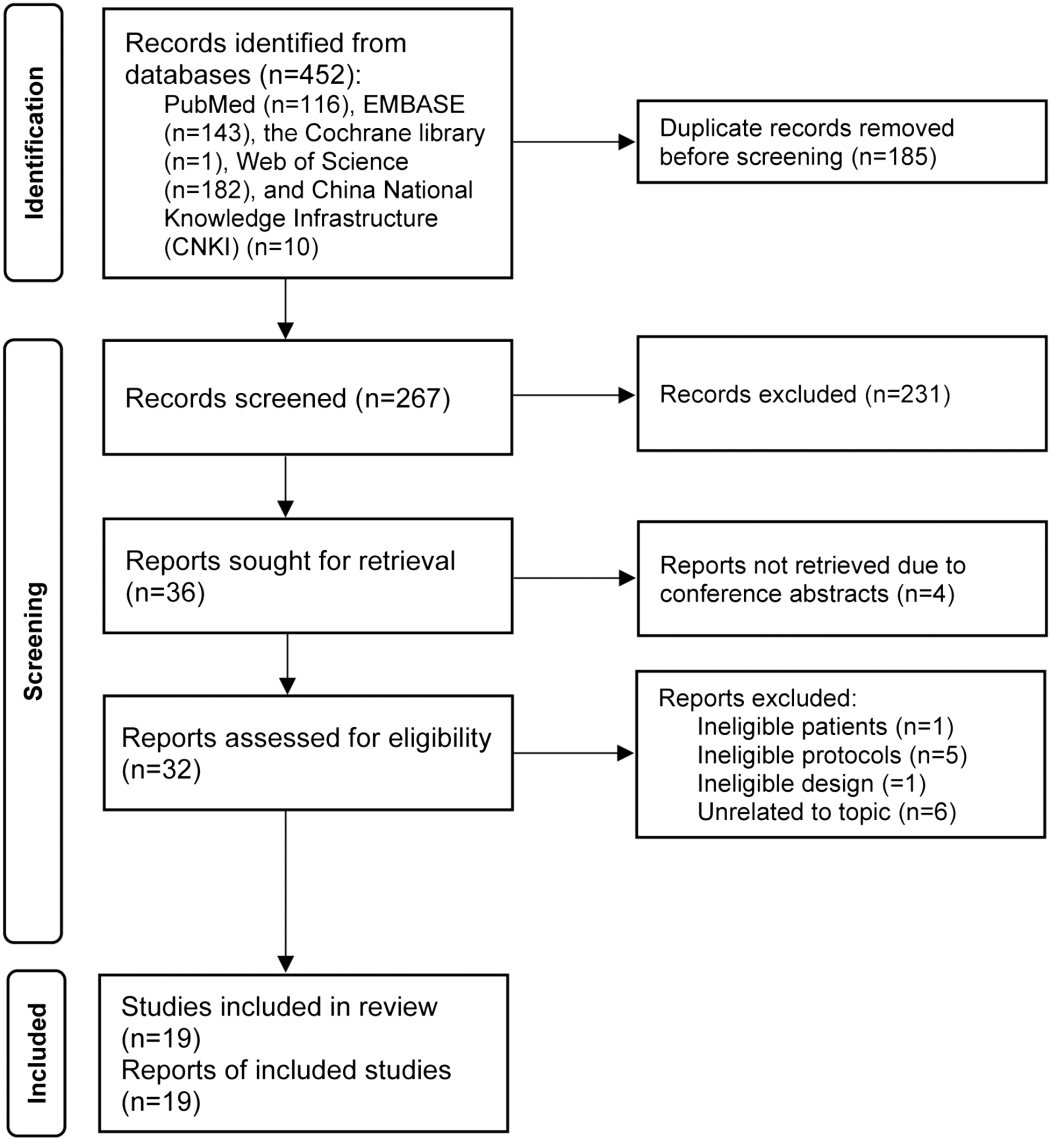
The PRISMA flow chart of study selection.

Electronic literature search initially identified a total of 452 relevant studies. After removing duplicate studies, 267 studies were eligible for the initial eligibility assessment. After screening titles and abstracts, we excluded 231 ineligible studies. Furthermore, four studies were excluded because they were conference abstracts. Finally, 19 studies (6, 12, 13, 19–34) were included in the final meta-analysis after excluding 13 ineligible studies due to: ineligible patients (*n* = 1), ineligible protocols (*n* = 5), ineligible study design (*n* = 1), and irrelevant to our topic (*n* = 6). The complete process of study selection and the corresponding reasons for excluding studies are shown in Figure 1.

### Baseline characteristics of eligible studies

A total of 1,257 patients from 19 studies conducted in 11 countries were included in this meta-analysis. Among 19 included studies, 16 studies applied contrast-harmonic model with SonoVue® (CHSV) (12, 13, 19–26, 28–30, 32–34), 2 studies applied contrast-harmonic model with Optison™(CHOS) (6, 27), and one study applied contrast-harmonic model with Lumason® (CHLS) (31). Seven studies (6, 20, 30–34) had a retrospective design and the others (12, 13, 19, 21–29) had a prospective. Eight studies (6, 13, 19, 24, 25, 29–31) recruited infants and the remaining 11 studies (12, 20–23, 26–28, 32–34) recruited both infants and adolescents as research subjects. The detailed baseline characteristics of all eligible studies are summarized in Table 1.

**Table 1:** Basic characteristics of 19 studies included in this network meta-analysis.

Study	Country	Study design	Sample size	Gender (M/F), *n*	Age	NKUU	VUR	Non-VUR	Indication for VUR investigation	Type of ceVUS	Contrast name
Ascenti et al., (19)	Italy	PS	80	36/44	3 months to 5 years	160	52	108	UTI, uretero-pelvic dilation, follow for VUR treatment	Contrast-harmonic model based on basic grey-scale and MI of ranging from 0.04–0.67	SonoVue®
Faizah et al., (21)	Malaysia	PS	27	17/10	1 month to 16 years	55	10	45	Antenatal pelvicalyceal dilation, UTI, neurogenic bladder, follow for VUR treatment	Contrast-harmonic model based on basic grey-scale	SonoVue®
Kis et al., (24)	Hungary	PS	183	94/89	2 days to 44 months	366	103	263	UTI, pelvicalyeal dilation, follow-up for VUR	Contrast-harmonic model based on basic grey-scale and MI of ranging from 0.4–0.6	SonoVue®
Ključevšek et al., (25)	Slovenia	PS	66	35/31	5 days to 1 year	132	16	116	Febrile UTI, bacteriuria, abnormal KUB ultrasound	Contrast-harmonic model based on basic grey-scale and MI of ranging from 0.06–0.1	SonoVue®
Mane et al., (26)	India	PS	30	21/9	1 month to 12 years	58	17	41	Febrile UTI, follow for VUR, neurogenic bladder	Contrast-harmonic model based on basic grey-scale	SonoVue®
Ntoulia et al., (27)	USA	PS	30	9/21	18 days to 17 years	62	12	50	Febrile UTI, bacteriuria, abnormal KUB	Contrast-harmonic based on basic grey-scale and MI of ranging 0.03–0.49	Optison™
Papadopoulou et al., (12)	Greece	PS	228	123/105	6 days to 13 years	463	71	392	UTI, follow-up of VUR, urinary tract dilation, sibling of child with VUR	Contrast-harmonic model based on basic grey-scale	SonoVue®
Piskunowicz et al., (28)	Poland	PS	83	46/37	1 month to 17.5 years	166	29	137	UTI, ureteral dilation, suspicion of reflux nephropathy	Contrast-harmonic model based on basic grey-scale	SonoVue®
Siomou et al., (29)	Greece	PS	60	44/16	2.2 months	123	12	111	Prenatal hydronephrosis	Contrast-harmonic model based on basic grey-scale	SonoVue®
Tang et al., (30)	China	RS	22	18/4	19 days to 1 year	44	4	40	Febrile UTI, postnatal hydronephrosis, multicystic dysplastic kidney	Contrast-harmonic model based on basic grey-scale	SonoVue®
Velasquez et al., (31)	USA	RS	39	20/19	31.9 months	84	17	67	Suspicion of UTI	Contrast-harmonic model based on basic grey-scale	Lumason®
Wong et al., (13)	China	PS	31	23/8	2 months to 4 years	62	14	48	UTI	Contrast-harmonic model based on basic grey-scale and MI of ranging from 0.05–0.07	SonoVue®
Woźniak et al., (32)	Poland	RS	80	18/62	3 months to 17.3 years	161	60	101	UTI, treated with VUR	Contrast-harmonic model based on color doppler (3D/4D) and MI	SonoVue®
Deng et al., (20)	China	RS	36	23/13	21 days to 10 years	72	51	21	Suspected VUR on US	Contrast-harmonic model based on color doppler and a MI of 0.08	SonoVue®
Fernández-Ibieta et al., (22)	Spain	PS	40	N/A	2 months to 13 years	80	34	46	Suspicion of VUR	Contrast-harmonic model based on basic grey-scale	SonoVue®
Woźniak et al., (32)	Poland	RS	69	21/48	1 year to 13.7 years	138	68	70	UTI, hydronephrosis	Contrast-harmonic model based on color doppler (2D/3D/4D) and MI	SonoVue®
Kim et al., (23)	Korea	PS	32	20/12	3 months to 16 years	63	27	36	Febrile UTI, suspicion of reflux nephropathy	Contrast-harmonic model based on basic grey-scale and MI of ranging from 0.07–0.1	SonoVue®
Paltiel et al., (6)	USA	RS	97	46/51	3 months	200	26	174	UTI, Prenatal hydronephrosis, solitary kidney	Contrast-harmonic model based on basic grey-scale and low MI	Optison™
Zou et al., (34)	China	RS	24	N/A	1 month to 14.5 years	48	23	25	Suspicion of VUR	Contrast-harmonic model based on color doppler and MI of ranging from 0.06–0.08	SonoVue®

PS, prospective study; RS, retrospective study; VUR, vesicoureteral reflux; UTI, urinary tract infection; NKUU, number of kidney-ureteral unit; ceVUS, contrast-enhanced voiding urosonography; N/A, not applicable.

### Risk of bias assessment

Detailed results of risk of bias assessment for all eligible studies are summarized in Supplementary Table S2. Regarding the risk of bias concerns, 84% (16/19) (12, 19–26, 28–34) did not clearly describe (unclear or high risk of bias) “patient selection” methods, 16% (3/19) (22, 32, 33) did not clearly describe detailed information for “index test and reference standard”, and 16% (3/19) (26, 32, 33) were judged as having high risk in the domain of “flow and timing” because there was not an appropriate interval between index test and reference standard. For applicability concerns, 90% (17/19) (32, 33) had low or unclear applicability concerns, except for two studies (32, 33) that had high applicability concerns on the index test.

### Evidence network

**Figure 2:**
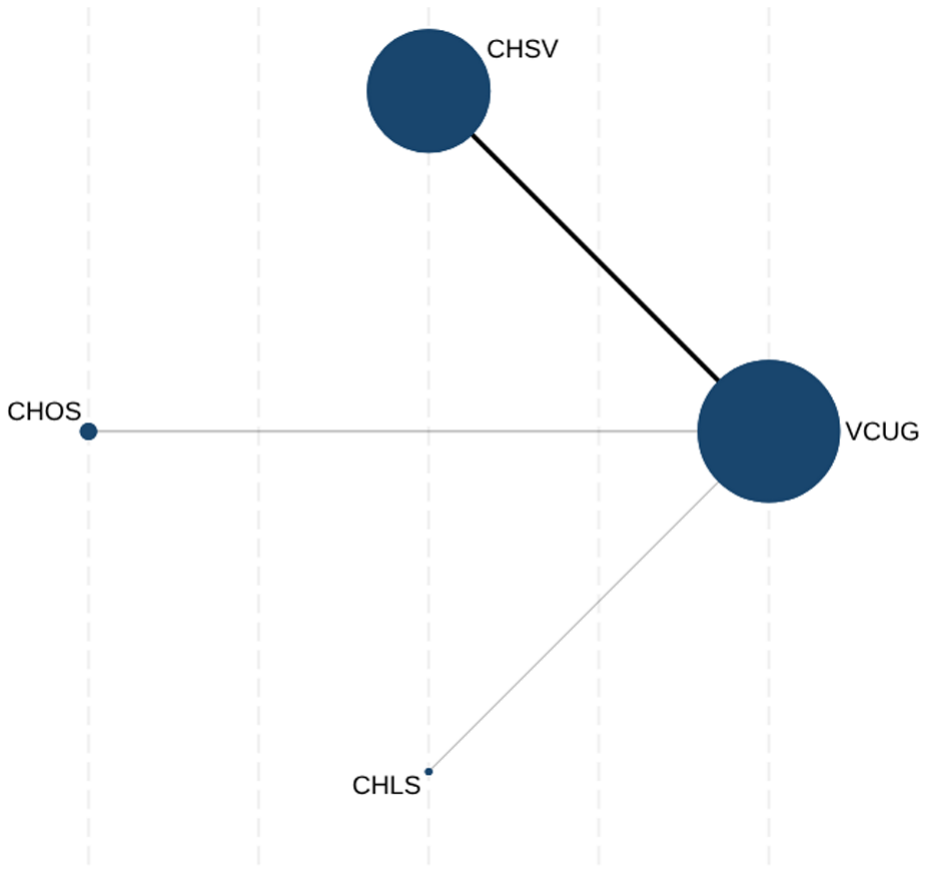
Evidence network of currently available ceVUS protocols. ceVUS, contrast-enhanced voiding urosonography; VCUG, voiding cystourethrography; CHSV, contrast-harmonic model with SonoVue®; CHOS, contrast-harmonic model with Optison™; CHLS, contrast-harmonic model with Lumason®.

Figure 2 shows the evidence structure for connecting all available diagnostic comginations of ceVUS imaging modalities with different second-generation contrast agents to VCUG. A solid black line connecting two protocols indicates a direct comparison between these two protocols, and the width of the line was weighted by the number of direct comparisons. A blue solid circle indicates a protocol, and the accumulated number of kidney-ureteral units weighted its size. As shown in Figure 2, more studies directly compared the diagnostic combination of CHSV with VCUG, and only limited studies focused on the diagnostic performance of CHOS and CHLS.

### Pairwise meta-analysis

**Figure 3:**
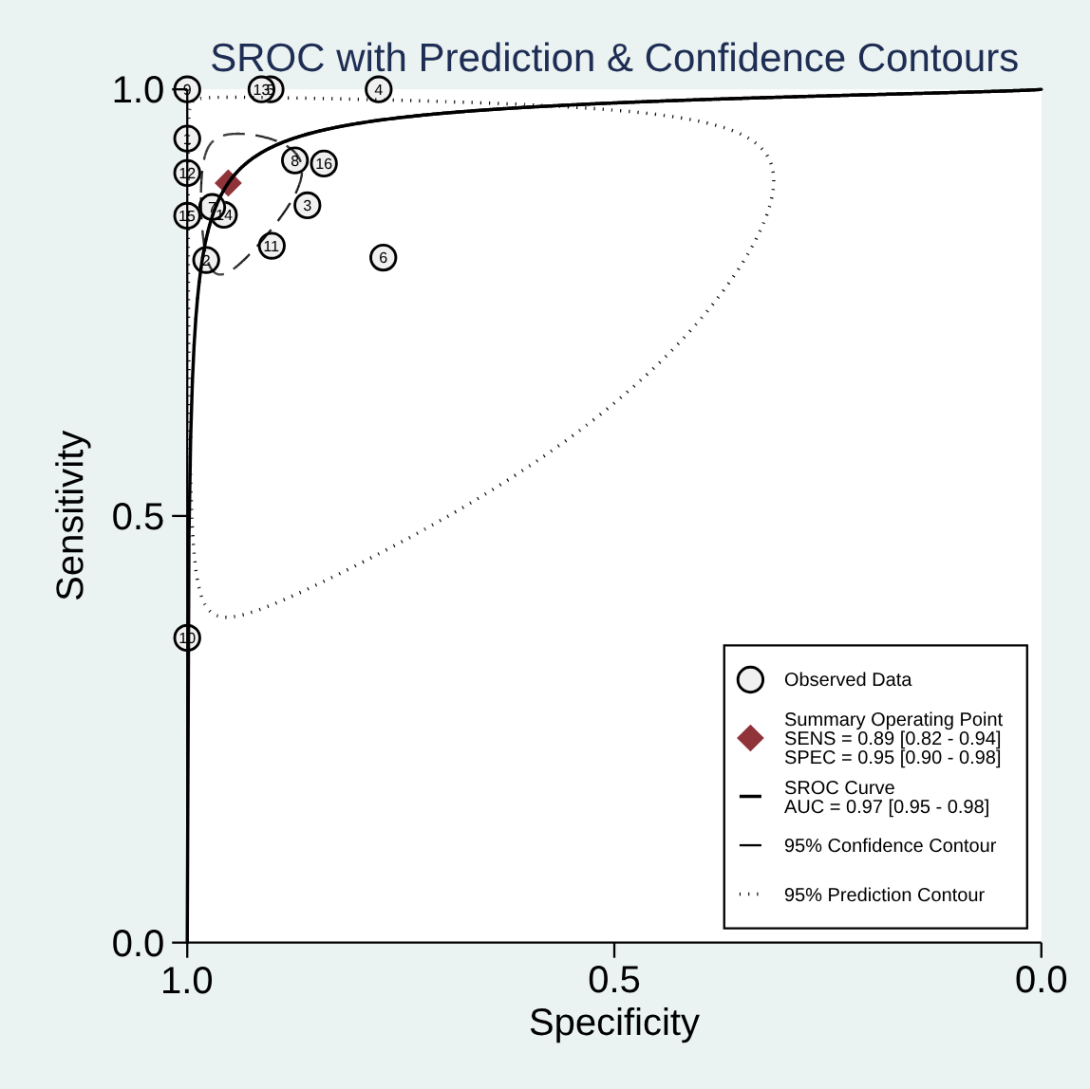
The pooled diagnostic performance of CHSV in detecting VUR. VUR, vesicoureteral reflux; SEN, sensitivity; SPE, specificity; PPV, positive predictive value; NPV, negative predictive value; DOR, diagnostic odds ratio; DA, diagnostic accuracy; AUC, area under the cumulative curve; CHSV, contrast-harmonic model with SonoVue®.

Figure 3 details the pooled diagnostic performances of CHSV, which shows strong effectiveness. The summary operating point indicates a sensitivity of 0.89 (95% CI: 0.82–0.94) and a specificity of 0.95 (95% CI: 0.90–0.98). The AUC of CHSV is 0.97 (95% CI: 0.95–0.98), further supporting the robust diagnostic ability of CHSV. CHOS and CHLS are represented by only 2 studies and 1 study, respectively. Due to the limited number of studies for these protocols, the HSROC is not applicable.

### Network meta-analysis results

As shown in Supplementary Figure S1, the sensitivity, specificity, and DA of CHSV were significantly worse than those of VCUG. However, the NPV of CHSV was higher than that of VCUG. For other comparisons, including CHSV, CHOS, CHLS, and VCUG, no statistical differences were found for all diagnostic measures.

### The probability of being best treatment option

We utilized SUCRA method to estimate the probability of being best for all available combinations of various ceVUS imaging modalities with different second-generation contrast agents. As shown in Table 2 and Supplementary Table S4, except for VCUG, CHSV had the probability of being best opting, with a probability of 50.9%, 16.6%, 42.6%, 57.4%, 36.2%, and 20.4% for SEN, SPE, PPV, NPV, DOR, and DA, respectively.

**Table 2:** SUCRA values of five different diagnostic strategies for VUR.

Diagnostic protocols	DA
VCUG (reference)	92.7%
CHSV	20.4%
CHOS	45.3%
CHLS	41.6%

SUCRA, surface under the cumulative ranking curve; VUR, vesicoureteral reflux; VUS, voiding urosonography; DA, diagnostic accuracy; CHSV, contrast-harmonic model with SonoVue®; CHOS, contrast-harmonic model with Optison™; CHLS, contrast-harmonic model with Lumason®.

### Publication bias

We inspected the risk of publication bias for CHSV, because only this diagnostic combination included more than five eligible studies. The plot for publication bias are shown in Supplementary Figure S2, and the asymmetry test did not achieve a significant level (*p* = 0.47) for CHSV.

## Discussion

The results of the current network meta-analysis suggested that the diagnostic combination CHSV had no satisfactory diagnostic performance because this protocol had significantly lower SEN, SPE, and DA than VCUG. However, CHOS might be a possible candidate for detecting vesicoureteral reflux in children and adolescents because it was comparable to VCUG in all diagnostic measures. More importantly, the results of SUCRA also revealed that the probabilities of CHOS in all diagnostic measures were only behind to VCUG, with a probability of being best of 50.9%, 16.6%, 42.6%, 57.4%, 36.2%, and 20.4% for Sen, Spe, PPV, NPV, DOR, and DA, respectively.

It has always been challenging to reduce the radiation dose while maintaining image quality when using radiation-based imaging techniques, particularly for younger patients more susceptible to ionizing radiation's adverse effects (23). An alternative to VCUG is ceVUS, an ionizing radiation-free technique for detecting VUR that utilizes ultrasound and a contrast agent administered into the bladder to image the urinary tract (6). Previous meta-analyses (7, 14) have confirmed the diagnostic performance of ceVUS in the detection of VUR in children and adolescents. However, these two meta-analyses did not separate the impact of different ceVUS imaging modalities and second-generation contrast agents on the overall diagnostic performance, thereby greatly limiting the reference value for clinical decision-making. In the current study, we firstly differentiate ceVUS imaging modalities, and also separately compare various diagnostic combinations of these available imaging modalities with different types of second-generation contrast agents. Subsequently, we assessed the synergistic diagnostic performance of various diagnostic combinations of different ceVUS imaging modalities with second-generation contrast agents in the detection of VUR in children and adolescents. In addition, we also ranked different diagnostic combinations by introducing the SUCRA method, therefore providing more useful information for clinical decision-making.

Our network meta-analysis suggests that ceVUS based on harmonic model using Optison™ may be the optimal diagnostic protocol for the detection of VUR in children and adolescents, which can be an alternative protocol to VCUG. Two types of second-generation contrast agents are available clinically, including SonoVue®/Lumason® and Optison™. SonoVue® is actually similar to Lumason®, and the difference between these two contrast agents is that they are used in different countries. Specifically, SonoVue® is primarily used in European countries, while Lumason® is primarily applied in the United States. However, Optison™ was approved for intravesical use in Canada, the United Kingdom, the European Union, India, Singapore, and China. In our analysis, the focus was deliberately placed on ultrasound technology rather than a direct comparison between contrast agents. This approach was chosen due to the inherent complexities and variabilities in ultrasound diagnostics, such as the experience of the clinician operating the ultrasound device and patient-specific characteristics, which can significantly influence diagnostic outcomes. These factors can act as confounders, affecting the reliability and consistency of results across different studies. It is hypothesized that different second-generation contrast agents have similar imaging properties (6); however, due to the lack of direct comparisons between them, it is impossible to demonstrate which type of second-generation contrast agents may be better than others. In the current network meta-analysis, we confirmed that Optison™ has better potential than SonoVue®/Lumason® because harmonic VUS using SonoVue®/Lumason® was inferior to VCUG, while harmonic VUS using Optison™ was comparable to VCUG in the detection of VUR in children and adolescents. It is noted that Optison™ have only been evaluated by limited studies (2 studies), so further assessing the comparative diagnostic performance of different second-generation contrast agents by performing larger studies directly comparing them is necessay.

With the advancement of technology and the provision of highresolution devices, three main ceVUS imaging modalities are currently available in clinical practice, including conventional, harmonic, and doppler-based imaging models. Up to date, there was no study has yet compared the difference between different imaging modalities. One previous meta-analysis showed that ceVUS using second-generation contrast with harmonic imaging achieved an excellent safety profile and acceptable diagnostic accuracy (14), revealing that harmonic imaging may be a preferred imaging modality for ceVUS. Harmonic imaging is primarily based on the non-linear propagation properties of ultrasound waves, increasing contrast and spatial resolution, helping obtain clearer and smoother images and detect more conspicuous microbubbles (35). Theoretically, the harmonic imaging model is better than the conventional imaging model and doppler-based imaging model. However, the current network meta-analysis did not find the difference between different ceVUS imaging modalities in their overall diagnostic performances because various combinations of different imaging modalities with the same contrast agents did not show statistical difference in all diagnostic measures. We speculate that the lack of difference in diagnostic measures across protocols may be primarily due to the use of different contrast agents rather than the imaging modalities themselves, which could explain the positive results observed in the previous meta-analysis that employed second-generation contrast agents.

The diagnostic performance of ceVUS in the detection of VUR may be affected by patient's age due to developmental variations during childhood (7). For example, one study revealed that ceVUS had the higher sensitivity in children aged less than 2 years than other age groups (36). However, the current network meta-analysis could not investigate the effect of patients' age on the diagnostic performance of various diagnostic combinations of different ceVUS imaging modalities with two types of second-generation contrast agents because eligible studies that enrolled children and adolescents did not separate these two types of patients into independent groups. Notably, we performed a transitivity assessment across comparisons based on patients' mean age, and the result showed that mean age was evenly distributed across comparisons, implying that our pooled results will not be significantly affected by patients' age. Nevertheless, we still suggest future studies to evaluate the impact of age on the diagnostic performance of different diagnostic combinations of different ceVUS imaging modalities with different contrast agents.

The current network meta-analysis had some methodological strengths to generate these promising findings. First, we firstly differentiated ceVUS imaging modality and contrast agents and evaluated various diagnostic combinations of different imaging modalities with contrast agents, which was benefical to more accurately determine the diagnostic performance of ceVUS in the detection of VUR in children and adolescents. Second, we firstly utilized network meta-analytic technique to compare various diagnostic combinations of different imaging modalities with contrast agents, which provided clinically important information because all these available diagnostic combinations have not yet been directly compared before. Third, the SUCRA method was used to rank all evaluated diagnostic combinations, which further provided detailed information about which diagnostic combination of different imaging modalities with contrast agents may have the probability of being best candidate for the detection of VUR in children and adolescents. Fourth, we conducted transitivity assessment before performing data analysis, therefore greatly enhancing the reliability of the pooled results.

We must also admit that the current network meta-analysis has certain limitations. First, we included 19 eligible studies for the final meta-analysis; however, most studies included only limited sample size, thus compromising the robustness of the pooled results. Specifically, only two studies evaluated 262 moieties regarding Optison™'s potentially better agreement rate with VCUG, which highlights the limited evidence and underscores the need for further validation with more extensive data. In fact, when we performed statistical heterogeneity examination, we found that three studies with relatively larger sample size were the contributor to the significant statistical heterogeneity because all statistical heterogeneity decresed to zero after these three studies were exluced from the corresponding analysis. High statistical heterogeneity can compromise the reliability of meta-analysis results by introducing variability that is not attributable to chance alone. By excluding the studies contributing to this heterogeneity, we achieved a more stable and accurate analysis, improving the overall credibility of our findings. By reviewing all eligible studies, we clearly known that studies with larger sample size generated more accurately estimates than other studies with limited sample size. Furthermore, This network meta-analysis divided protocols into four groups, which may have impacted the statistical power. Therefore, it is necessary to perform more studies with large-scale to further evaluate the diagnostic performances of different diagnostic combinations of different ceVUS imaging modalities with different contrast agents. Second, most studies included in this network meta-analysis only directly compared the diagnostic performance of harmonic VUS using SonoVue® or Lumason® with VCUG for the detection of VUR; however, only limited studies compared other diagnostic combinations of different ceVUS imaging modalities and contrast agents with VCUG. Therefore, the diagnostic performance for these diagnostic combinations should be interpreted with caution. Third, this study adopted VCUG as the diagnostic gold standard despite its associated radiation exposure. However, it is important to acknowledge that the diagnostic accuracy of VCUG may also have certain limitations. In contrast, ceVUS offers a less invasive alternative with minimal radiation risk. Therefore, this study was conducted to explore which ceVUS protocol achieves diagnostic accuracy closest to that of VCUG, providing valuable guidance for clinical practice. The reflux detected on ceVUS alone and absent on VCUG has been shown to be true positive. Many comparative studies have shown that one detects on average about 10% more reflux on ceVUS than on VCUG. Future studies are expected to compare the results of all positive VCUG and ceVUS with each outcome. Fourth, there were significant variabilities in ceVUS protocols and VCUG between included studies, which also negatively affected the pooled results. Fifth, seven eligible studies used a retrospective design, inevitably biased the results. Sixth, the methodological strategies of network meta-analysis for diagnostic test accuracy are still not well developed, it may need further updating. Seventh, this study did not include an analysis of reflux grading due to variability in grading criteria across the studies and the lack of consistent data, and further research is needed to address this aspect. Finally, we detected the risk of publication bias for the meta-analysis of conventional VUS using SonoVue®/Lumason®. In addition, publibication bias examination was not performed for harmonic VUS using Optison™ and doppler-based VUS using SonoVue®/Lumason® due to limited eligible studies. So, the pooled results of these three diagnostic combinations may be negatively affected by publication bias. Despite these limitations, we believe that the results of the current network meta-analysis provide a useful reference for clinical practitioners to choose a reasonable diagnostic combination of different ceVUS imaging modalities and second-generation contrast agents for VUR detection.

## Conclusions

In conclusion, the results of the current network meta-analysis show that compared with other ceVUS methods, ceVUS based on harmonic imaging modality using Optison™ may be a better diagnostic combination for the detection of VUR in children and adolescents. However, potential diagnostic misses and limitations such as sample size and study design variations, which could impact the efficacy of diagnostic methods in clinical settings, and more studies with larger sample sizes are needed to further validate our findings.

## Data Availability

The original contributions presented in the study are included in the article/Supplementary Material, further inquiries can be directed to the corresponding authors.
